# Clinical value of color doppler ultrasound in prenatal diagnosis of umbilical cord entry abnormity

**DOI:** 10.12669/pjms.325.10518

**Published:** 2016

**Authors:** Jiandong Sun, Li Wang, Yinghui Li

**Affiliations:** 1 Jiandong Sun, Department of Special Inspection, Binzhou People’s Hospital, Shandong, 256600, China; 2 Li Wang, Department of Special Inspection, Binzhou People’s Hospital, Shandong, 256600, China; 3 Yinghui Li, Department of Health Examination, Binzhou People’s Hospital, Shandong, 256600, China

**Keywords:** Umbilical cord entry abnormity, Ultrasonic diagnosis, Second trimester

## Abstract

**Objective:**

To study the clinical value of prenatal diagnosis of umbilical cord entry abnormity (UCEA) by means of color Doppler ultrasound (CDUS).

**Methods:**

Clinical data of sixty-four cases with confirmed umbilical cord entry abnormity were reviewed and the specific UCEA conditions and the outcomes of perinatal infants were analyzed.

**Results:**

Detection rates of marginal umbilical cord entry abnormity and velamentous umbilical cord entry abnormity by means of CDUS at second trimester were 94.1% and 93.8% respecdtively much higher than 80.0% and 68.8% which were those of third trimester. Discrepancy had statistical significance (P<0.05). True positive rate of prenatal diagnosis of UCEA by means of CDUS was 85.9% (55/64), and false negative rate was 14.1% (9/64). Among sixty four patients with UCEA, seventeen patients (26.6%) underwent selective caesarean delivery; twenty-six patients (35.9%) underwent emergency caesarean delivery and twenty-four patients (37.5%) had normal delivery.

**Conclusion:**

Prenatal diagnosis of UCEA by means of CDUS is intuitive and accurate. It provides an evidence for determination of the best time to diagnose UCEA, and also offers a proper advice for pregnant women about delivery mode to ensure the fetus survival rate, which is clinically valuable.

## INTRODUCTION

Umbilical cord entry abnormity (UCEA) includes marginal umbilical cord entry abnormity (MUCEA) and velamentous umbilical cord entry abnormity (VUCEA).^1^ It is known that marginal umbilical cord entry, namely battledore placenta, accounting for 7% of all is commonly seen. In this case, umbilical cord entry locating within 2cm of the placental edge has no effects on mothers and children. Therefore, it has no clinical values.^2^,^3^ Velamentous umbilical cord entry locates at free embryonic membrane outside the placental edge, and umbilical vein twists through amnion and chorion and then goes deep in the placenta.^4^ Without the protection of wharton jelly, the rupture and embolization of umbilical vessels are more likely to happen. When pregnant women with velamentous umbilical cord entry give birth, rupture of umbilical vessels owning to uterine contractions and other reasons, will lead to the fetus death. Hence, VUCEA is a disease seriously threatening the safety of fetus and the disease is related to infants with low birth weight, small for gestational age, and preterm birth.^5^,^6^

Nowadays, the main method to diagnose UCEA is color Doppler ultrasound (CDUS). Pregnant women often undergo prenatal screening at second trimester and third trimester.^7^ When pregnant women with UCEA give birth, UCEA, especially VUCEA, dilates cervix, compresses blood vessels of the fetus, and even threatens his life.^8^,^9^ Therefore, during antenatal examination, attention should be paid at placenta and umbilical cord condition. Once UCEA occurs, corresponding measures should be taken to solve the problem, for example, pregnant women, who are diagnosed as velamentous umbilical cord entry, or combined with vasa previa through CDUS, can choose cesarean delivery.^10^ This research reviewed and analyzed the clinical data of sixty-four cases with confirmed UCEA by means of CDUS to study the clinical value of prenatal diagnosis of UCEA by means of CDUS and provide a reference for the effective prenatal diagnosis of UCEA.

## METHODS

### General data

Sixty-four pregnant women who were confirmed with confirmed umbilical cord entry abnormity in Binzhou People’s Hospital, from March, 2013 to March, 2015 were chosen for the research. All patients gave informed consent for the study. Among them, forty-one cases were primipare, twenty-three cases were multipara, and both were singleton pregnancy. Their ages varied from 20 to 35.4 years old and the average age was 25±2.23 years old. Their weights varied from 45 to 67 kg and the average weight was 49.8±5.5 kg. Their duration of pregnancy varied from 18 to 41 weeks and the average duration of pregnancy was 27±2 weeks. Thirty two pregnant women were confirmed with marginal umbilical cord entry, among which seventeen were at second trimester and fifteen were at third trimester. Thirty-two pregnant women were confirmed with velamentous umbilical cord entry, among which sixteen were at second trimester and sixteen were at third trimester.

### Instruments and methods

GE Voluson E6 color doppler ultrasonic diagnosis apparatus (General Electric Company, Fairfield City, Connecticut, USA) with 3.5 ~ 5.0 MHz Convex array probe and 4. 0 ~ 7.0 MHz volume probe was used. Pregnant women lied in the supine position to make abdomen exposed. After routine examination, an overall check for placenta, umbilical cord and appendix was conducted. The whole placenta was scanned. If the placenta could not be seen clearly, doctors could ask pregnant women to walk or change the position, and then scanned the umbilical cord, found the insertion at the placenta, and examined the changes by means of CDUS. The whole placenta should be fully exposed when diagnosing by means of CDUS, which helped to find the umbilical cord entry. The insertion point especially needed detailed examination, for example, doctor could circulate 360 degrees to check the umbilical cord. Close look was required because MUCEA might not be detected in a certain plane. In that case, a full bladder helped in the examination. Doctor could also use two dimensional images from CDUS to observe the insertion point of vessels and measured the length between the insertion point and the edge of placenta. Results of prenatal diagnosis of UCEA by means of CDUS and results of pathological diagnosis were compared and analyzed.

### Diagnostic criteria

(1) Diagnostic basis of MUCEA was that the length between insertion point and the edge of placenta was less than 2cm.

(2) Velamentous placenta: diagnostic basis of complete velamentous placenta was that there was no insertion point at the surface of placenta, and branches of umbilical cord were at fetal membrane; diagnostic basis of partial velamentous placenta was that the insertion at the surface of placenta had several branches, most of which went deep into the placenta, and a few of which were at fetal membrane.

### Statistics analysis

All data were input and analyzed by SPSS ver. 20.0. Enumeration data were examined by chi-square test and P<0.05 meant that difference had statistical significance.

## RESULTS

### Image analysis

**Fig.1:**
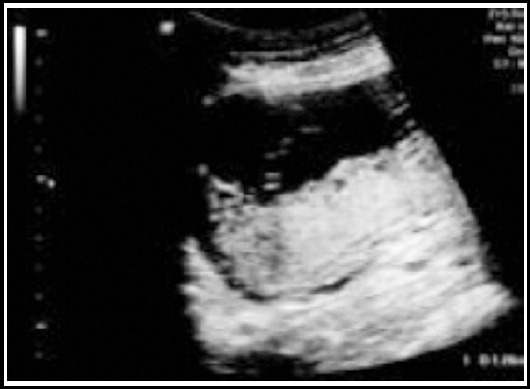
Marginal umbilical cord entry.

**Fig.2:**
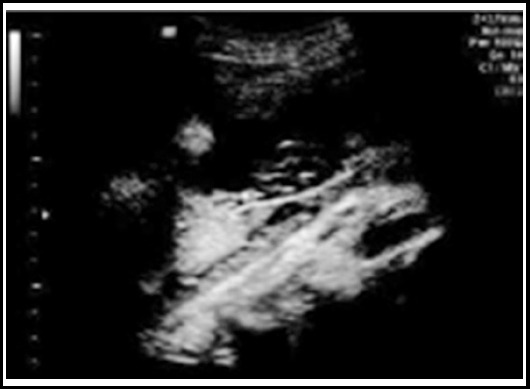
Velamentous umbilical cord entry.

Vascular structure, a tree structure connecting branches of umbilical cord vessels and attachment points in the placenta, could be clearly seen by CDUS. When umbilical cord attaches at the edge of placenta, which is racket-shaped or fan-shaped, and the length between insertion and the edge of placenta is less than 2cm, it is called marginal umbilical cord entry. When the root of umbilical cord and fetal membrane are attached mutually; there is no insertion at the placenta, and three umbilical cord vessels starts from insertion through amnion and chorion, finally deep into the placenta, it is called velamentous insertion of umbilical cord. Details are shown in (Fig. 1 and 2).

### Comparison of detection rates of UCEA at different gestation periods

Sixteen cases were diagnosed as MUCEA at second trimester, detection rate of which was 94.1%. Twelve cases were diagnosed as MUCEA at third trimester, detection rate of which was 80.0%. Detection rate at second trimester was much higher than that of third trimester (P<0.05). Fifteen cases were diagnosed as VUCEA at second trimester with a detection rate of 93.8%. Eleven cases were diagnosed as MUCEA at third trimester with a detection rate of 68.8%. Detection rate at second trimester was much higher than that of third trimester (P<0.05). (Table-I).

**Table-I:** Comparison of detection rates of UCEA at different gestation periods [N (%)].

Gestation period	MUCEA(N=32)		VUCEA(N=32)	

	Detected	Not detected	Detected	Not detected
Second trimester	16(94.1)	1(5.9)	15(93.8)	1(6.2)
Third trimester	12(80.0)	3(20.0)	11(68.8)	5(31.2)
X^2^	5.172		6.524	
P	P<0.05		P<0.05	

### Comparison between ultrasonic diagnosis and pathological diagnosis of UCEA

Among sixty-four patients with confirmed UCEA, fifty-five cases with UCEA were diagnosed by CDUS, and true positive rate was 85.9%; there were nine cases of missed diagnosis, and false negative rate was 14.1%. Sixty-four cases were all pathologically diagnosed, and true positive rate was 100%; there was no missed diagnosis, and false negative rate was 0.0%.

### Outcomes of perinatal infants with UCEA

Among sixty-four perinatal infants with UCEA, seventeen patients underwent selective cesarean delivery (26.6%), twenty three patients underwent emergency cesarean delivery (35.9%), and twenty-four patients underwent natural labor (37.5%). Table-II.

**Table-II:** Outcomes of sixty-four perinatal infants with UCEA [N (%)].

Category of UCEA	Selective cesarean delivery	Emergency cesarean delivery	Natural delivery
MUCEA (N=32)	12(37.5)	3(9.4)	17(53.1)
VUCEA (N=32)	5(15.6)	20(62.5)	7(21.9)
Summation	17(26.6)	23(35.9)	24(37.5)

## DISCUSSION

Umbilical cord bridges between matrix and fetus. The passage from umbilical cord to placenta makes it possible to exchange nutrients and metabolite.^11^ Therefore, if umbilical cord has abnormity, it can threaten the life of mother and child. Abnormal umbilical cord insertion, including MUCEA and VUCEA, is clinically commonly seen.^12^,^13^

Abnormal umbilical cord insertion has few clinical features. Marginal umbilical cord entry leads to a small number of abnormal gestation and birth, while velamentous insertion of umbilical cord during pregnancy results in growth retardation, premature delivery, fetal hemorrhage, and higher risk of death.^14^ Clinically, the rate of cesarean delivery is much higher than that of natural birth^15^, which is similar to the results of this research. The discovery of MUCEA helps to find the problems of early fetal development, decreases the rate of stillbirth, premature birth, premature rupture of membrane, and raises the survival rates of fetus.^16^ Therefore, it is extremely significant to determine the best time to diagnose UCEA.

The results of this research showed that among thirty-two cases with confirmed MUCEA, detection rate of UCEA by means of CDUS at second trimester was 94.1%, much higher than 80.0% at third trimester (P<0.05). Among thirty-two cases with confirmed VUCEA, detection rate of UCEA by means of CDUS at second trimester was 93.8%, much higher than 68.8% at third trimester (P<0.05). For whether pregnant women with MUCEA or with VUCEA, detection rate at second trimester was higher than that at third trimester, the reason being that second trimester had high level of amniotic fluid, fetal movements were not limited, observation space was adequate and it was easy to trace the trend and insertion of umbilical cord. At third trimester, amniotic fluid was less, fetus bigger, observation space smaller, which on the one hand, made it hard to trace the insertion of umbilical cord, and on the other hand, influenced the clarity of the image from CDUS, causing difficulty in determining the umbilical cord entry.^17^,^18^ From eighteen to twenty-eight weeks of pregnancy usually is the best time to diagnose UCEA. Pregnant women should avoid ultrasonic diagnosis at third trimester, but overall examination was also needed. Improving diagnostic accuracy is vital for diagnosing UCEA and increasing survival rate of fetus.^19^

This research compared ultrasonic diagnosis and pathological diagnosis of UCEA. The results showed that fifty-five cases with UCEA were diagnosed out by CDUS, and true positive rate was 85.9%; there were nine missed diagnosis, and false negative rate was 14.1%. Sixty-four cases were all pathologically diagnosed, and true positive rate was 100%; there was no missed diagnosis, and false negative rate was 0.0%. It could be seen that true positive rate of diagnosis of UCEA was relatively high. Abnormal manifestation of MUCEA was that umbilical cord going deep into the placenta located within 2 cm of the edge of placenta, in which case, red artery and blue vein gathered around the placenta. Abnormal manifestation of VUCEA was that several thick vessels floating around membrane pull the membrane and there was no insertion point at the placenta. Entangled flow signals were attached at free membrane and fetus. The insertion at the surface of placenta had several branches, most of which went deep into the placenta, and a few of which were at fetal membrane.

### Limitations of the study

A previous study^20^ suggested that, different types of umbilical abnormality could induce dead fetus. But in this study, the survival rate of fetus was 100%, which was a quite positive outcome. It might be correlated to the small sample size.

## CONCLUSION

In conclusion, clinical value of diagnosing UCEA by CDUS lies in increasing diagnostic rates of UCEA, and it suggests a great guiding significance for monitoring fetal situation and choosing the delivery mode. Using CDUS to diagnose UCEA at second trimester can increase diagnostic rate. Moreover, this diagnostic method is simple and convenient, does little damage to mothers and fetus, and also economical. All factors make the method worth clinical promotion.
